# AOP-networkFinder—a versatile tool for the reconstruction and visualization of Adverse Outcome Pathway networks from AOP-Wiki

**DOI:** 10.1093/bioadv/vbaf007

**Published:** 2025-01-22

**Authors:** Nurettin Yarar, Marvin Martens, Torbjørn Rognes, Jan Lavender, Hubert Dirven, Karine Audouze, Marcin W Wojewodzic

**Affiliations:** Department of Informatics, University of Oslo, Oslo 0373, Norway; Department of Chemical Toxicology, Norwegian Institute of Public Health, Oslo 0456, Norway; Department of Bioinformatics (BiGCaT), NUTRIM, Faculty of Health, Medicine and Life Sciences, Maastricht University, Maastricht 6229, The Netherlands; Department of Informatics, University of Oslo, Oslo 0373, Norway; Department of Microbiology, Oslo University Hospital, Oslo 0424, Norway; Department of Computer Science, University of East Anglia, Norwich NR47TJ, United Kingdom; Department of Chemical Toxicology, Norwegian Institute of Public Health, Oslo 0456, Norway; University Paris Cité, Paris 75006, France; Department of Chemical Toxicology, Norwegian Institute of Public Health, Oslo 0456, Norway; Department of Research, Cancer Registry of Norway, Norwegian Institute of Public Health, Oslo 0456, Norway

## Abstract

**Motivation:**

The Adverse Outcome Pathways (AOP)-Wiki, a knowledge database for AOPs, requires an efficient way to present an overview of its content for the reconstruction of networks by experts in a given domain. We have developed the AOP-networkFinder, a user-friendly tool that retrieves AOPs of interest, allows network generation and cleaning, and finally visualizes networks built around the retrieved AOPs. Our tool constructs AOP networks by connecting AOPs that use the same Key Events (KEs) in a versatile but controlled manner. Genes related to these KEs are also displayed. The constructed networks can then be exported as images or to Cytoscape for further fine-tuning and statistical analysis.

**Results:**

The AOP-networkFinder allows users to comprehensively identify relationships between KEs and visualize the overall structure of an AOP both quickly and easily. This is immensely beneficial to researchers who need to understand the complex interplay between different KEs and the overall pathway they are studying and helps them to build further networks of interest while logging relevant information about changes within the network. These efforts are in line with the Findable, Accessible, Interoperable, and Reusable principles, which are crucial attributes for any developed databases and tools for optimizing (re)use in a dynamically changing landscape of AOP-Wiki.

**Availability and implementation:**

The AOP-networkFinder is an open-source application and is available online at aop-networkfinder.no, in the ‘Computational Toxicology at Norwegian Institute of Public Health’ Zenodo community at DOI 10.5281/zenodo.11068434, in the GitHub repository at github.com/folkehelseinstituttet/AOPnetworkFinder_v1, as well as in a Docker image at hub.docker.com/r/nurre123/aop_network_finder. The software is available under the GNU Affero General Public License (AGPL), v3.0. The tool uses the AOP-Wiki SPARQL endpoint to retrieve AOP data.

## 1 Introduction

The realm of human and environmental toxicology and risk assessment has witnessed significant advancements through the conceptual framework of Adverse Outcome Pathways (AOPs) (Ankley *et al.* 2010). An AOP describes a linear sequence of biological knowledge linking a Molecular Initiating Event (MIE) to an Adverse Outcome (AO) through intermediate Key Events (KEs) at several levels of the biological organization upon stressor exposure. The AOP framework, endorsed by the Organisation for Economic Co-operation and Development (OECD), offers a dynamic understanding of event linkage, via Key Event Relationships (KERs) in an acyclic graph (Villeneuve *et al.* 2018). The AOP concept holds significant importance in Next Generation Risk Assessment and the development of New Approach Methodologies (NAM), serving as a cornerstone in the regulatory landscape. Since AOPs provide a structured framework that links molecular-level perturbations to AOs at higher levels of biological organization, they facilitate the use of mechanistic data in regulatory decision-making. For example, AOPs have been employed in NAM development to predict skin sensitization, where mechanistic insights are used to reduce the reliance on animal testing (Aleksic *et al.* 2024).

Currently, only one central repository exists; the AOP-Wiki (aopwiki.org/). The AOP-Wiki, coordinated by the OECD and maintained primarily by JRC and the US EPA, is a collaborative platform where scientists, stakeholders, and regulators can contribute to the development and international sharing of AOPs.

Biological events (MIE, KE, and AO) are rarely exclusive to a single AOP, reflecting biological complexity and real-world scenarios. Where events are shared by several AOPs, it is possible to reconstruct AOP network (Knapen *et al.* 2018, Villeneuve *et al.* 2018). AOP networks, facilitated by KERs as the functional units, not only reveal gaps within existing networks but also illuminate critical pathway leading to AOs (Knapen *et al.* 2018, Villeneuve *et al.* 2018). For example, the CIAO project (ciao-covid.net/) demonstrated the interconnected nature of AOPs and their application to address complex challenges such as those posed by the COVID-19 pandemic (Clerbaux *et al*. 2022). The final AOP network showed a complete picture of known paths toward adversities and highlighted hub KEs through which many AOPs propagated to their AO.

The evolution of the AOP framework has embraced the web of linked data through the use of Resource Description Framework (RDF). RDF serves as the backbone for representing AOP information, can be explored using the SPARQL query language, and is an attractive concept for seamlessly integrating diverse databases. Tools such as the AOP-Wiki RDF allow accessing AOP-Wiki contents computationally and in a flexible, reproducible manner. However, these tools require knowledge of the SPARQL query language to explore RDF, necessitating user-friendly solutions for broader accessibility (Martens *et al.* 2022).

Despite its great potential, the complexity of RDF raises concerns about user-friendliness and accessibility, prompting the need to bridge the gap between RDF formats and more user-friendly tools. This need becomes especially evident in interdisciplinary projects like the Partnership for the Assessment of Risks from Chemicals (PARC), where the urgency for embracing enhanced connectivity and usability was required for the creation of an inventory of relevant existing AOPs and associated AOs from AOP-Wiki, where the AOP-Wiki RDF was utilized (Jaylet *et al.* 2024).

Several tools and web services have been developed to help the construction and exploration of AOPs and AOP networks such as the AOPWIKI-explorer, the sAOP or third-party tools from AOP-Wiki, including AOP-helpFinder, Biovista Vizit, Wiki-Kaptis, etc (Supplementary Table S1). As of today, about 500 AOPs have been proposed, presenting new challenges such as the need to make AOPs more Findable, Accessible, Interoperable, and Reusable (FAIR) (Wilkinson *et al.* 2016), more quantitative, and to utilize AOP networks more effectively. Tools facilitating further data integration are expected to play a crucial role in meeting these future needs.

**Figure 1.**
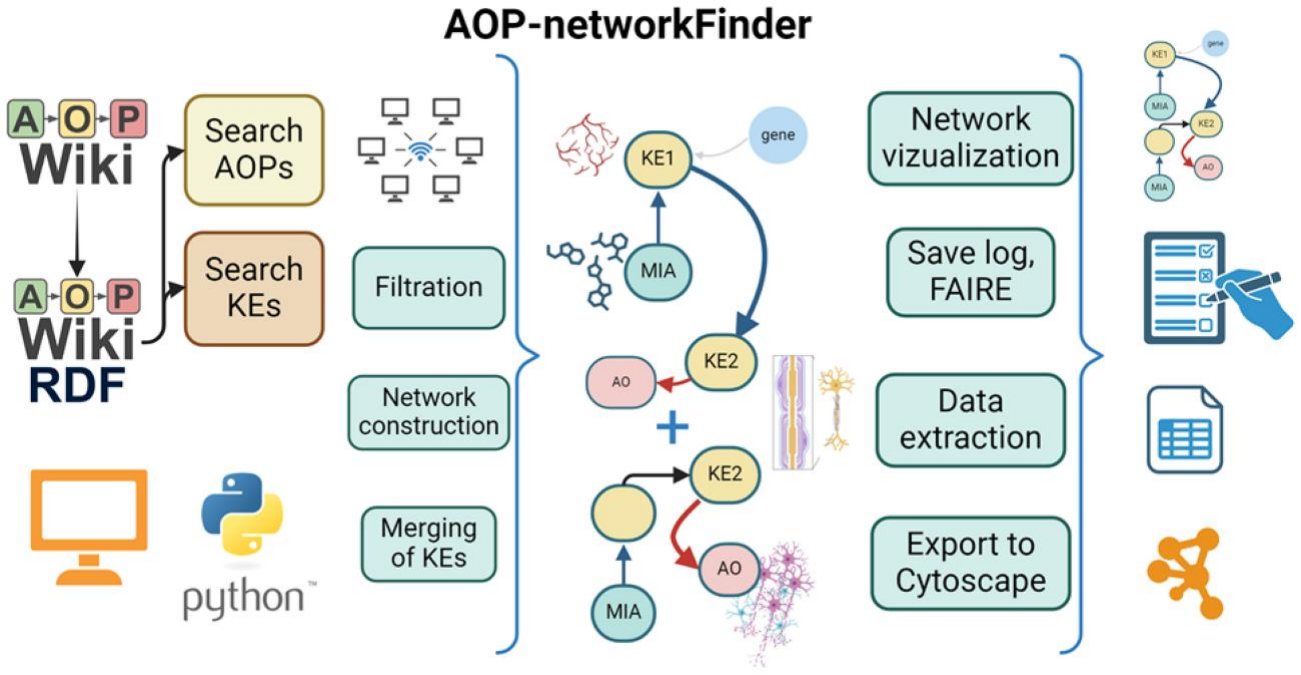
Concepts of data retrieving from AOP-Wiki via RDF format, and main functionalities of AOP-networkFinder tool. Created with Biorender.com.

Here we propose a user-friendly tool, named AOP-networkFinder, that reconstructs AOP networks from the AOP-Wiki. AOP-networkFinder uses the RDF representation of its contents, and addresses the FAIR principles, particularly in enhancing the accessibility of the AOP-Wiki data (Fig. 1). By allowing for automatic extraction and visualization, this tool proves itself both novel and complementary to the pre-existing ones. It alleviates researchers’ workload and contributes to the advancement of AOP-related research, innovation and applications.

The AOP-networkFinder is an open-source application with a graphical interface to explore and manipulate AOP networks, and is freely available online at aop-networkfinder.no

## 2 Methods

### 2.1 AOP-networkFinder

The AOP-networkFinder was crafted in Python3. The foundation is laid on Flask (version 3.0.0). The essential packaging tools, pip (v. 21.2.4) and wheel (v. 0.37.0) streamline the management and distribution of dependencies. Templating is achieved through Jinja2 (v. 3.1.2) and MarkupSafe (v. 2.1.3), enhancing the tool’s flexibility and rendering capabilities. The dynamic aspects are handled by Werkzeug (v. 3.0.1), while click (v. 8.1.7) facilitates command-line interface creation. AOP-networkFinder also employs Blinker (v. 1.7.0) for signal dispatching and ItsDangerous (v. 2.1.2) for secure token generation and validation. The comprehensive set of supporting libraries includes: NetworkX, SPARQLWrapper, pyparsing, pandas, pytz, numpy, python-dateutil, six, openpyxl, and textdistance. To automatically leverage the contents of the AOP-Wiki in a flexible and reproducible manner, AOP-networkFinder employs dynamic SPARQL queries against the AOP-Wiki’s SPARQL endpoint that contains knowledge graphs in RDF format (Martens *et al.* 2022).

### 2.2 Functionality of the tool

The primary functionality revolves around extracting and utilizing the AOP-Wiki’s data for efficient AOP network construction by means of SPARQL queries against the AOP-Wiki SPARQL endpoint (aopwiki.rdf.bigcat-bioinformatics.org). Automation replaces the manual process of combing through the AOP-Wiki and creating visualizations by offering features for graph manipulation and comprehensive, anonymous logging of user interactions. This makes the AOP-networkFinder tool most fitting for AOP network construction and manipulation (Supplementary Table S1). This ensures reproducibility and tracks changes made to the graph. AOP construction utilizes Object-Oriented Programming for modular encapsulation, promoting ease of reuse.

#### 2.2.1 Multi-search of AOPs and KEs

Addressing the AOP-Wiki’s limited search capabilities, the tool automates the process of extracting and constructing AOP networks based on user-specified IDs (Supplementary Fig. S1A). Utilizing AOP and KE encapsulation, the tool iterates through lists to produce visual representations of AOP networks when sharing common KEs. The tool avoids duplicates and automatically updates KE objects, streamlining the network-building process. By double-clicking on the node the tool will display detailed information about this node (Supplementary Fig. S1B). In addition, highlighting of a chosen AOP is possible.

#### 2.2.2 Filtering for AOPs and merging of KEs

Recognizing the need for filtering endorsed AOPs by the OECD, the tool allows users to filter AOPs based on their status (Supplementary Fig. S1A). Additionally, to counter potential redundancy from users introducing functionally similar KEs, the AOP-networkFinder compares KE names at runtime utilizing an artificial intelligence (AI)-based algorithm, the Levenshtein distance (Levenshtein 1965). Using fuzzy logic, if names are similar, users are prompted to merge KEs, fostering discussion among experts and accurate biological representation.

#### 2.2.3 Displaying of nearest neighbour from given KE

The tool can display the closest neighbours to given KEs. The network can be set to show points at a distance of either one or two steps (degree 1 or 2) from the chosen KEs (Supplementary Fig. S1C). This allows the user to better visualize the connections and relationships that the selected KEs have with other KEs, AOPs, and relevant genes, which are based on text-mapping in KE descriptions in the AOP-Wiki.

#### 2.2.4 Exporting graphs and logging user actions

While the AOP-networkFinder does not intend to analyse the overall network structure, it allows the user to export graphs compatible with Cytoscape for downstream analysis (Shannon *et al.* 2003). The tool logs every user action, including modifications made to the AOP networks, aiding in the reproducibility and validation of network changes. The logs capture all interactions, ensuring transparency and traceability of user-driven modifications, which can be used to validate the biological plausibility of changes. As the AOP-Wiki evolves, the log file helps users generate current versions of AOP networks. The tool allows the user to download the logs from the user interface, ensuring that all edits are documented for future reference and validation, and these are not stored elsewhere.

#### 2.2.5 AOP extraction and exporting raw data

The tool can extract raw AOP-Wiki data in a table format, offering users the option to export to ‘csv’ or ‘xlsx’. AOP-networkFinder stands out by eliminating the need for prior RDF knowledge and providing simplicity and user-friendly functionality (Supplementary Fig. S1D).

#### 2.2.6 Extracting author information

The AOP-networkFinder can also extract author information, as a crucial aspect under the BY-SA license is to credit the original authors of the AOP and KE content. The tool adds the original authors to the log to allow users to credit the original AOP creators.

## 3 Results

### 3.1 Case study of neurotoxicity AOPs: merging KEs for improved network representation

The capabilities of the AOP-networkFinder tool were demonstrated with a case study of neurotoxicity, focussing on AOP 260 ‘CYP2E1 activation and formation of protein adducts leading to neurodegeneration’ and AOP 12 ‘Chronic binding of antagonist to N-methyl-D-aspartate receptors (NMDARs) during brain development leads to neurodegeneration with impairment in learning and memory in aging’. The retrieval of both AOPs presented the AOs as separate entries as they had different names and showed the AOPs as unconnected as they shared no KEs. However, the tool identifies potential similarities in KEs (based on low Levenshtein distance) and prompts the user to merge them. In this case study, the tool suggests merging the AOs ‘N/A, Neurodegeneration’ and ‘Neurodegeneration’ (Supplementary Fig. S1). This process is designed to consolidate functionally similar KEs, enhancing the biological representation of AOPs. After merging the AOs, AOPs 12 and 260 now share the same AO node, ‘Neurodegeneration’, transforming the linear AOPs into an interconnected AOP network.

Upon opting to export the graph to Cytoscape, users can leverage Cytoscape’s extensive functionalities for network exploration. Finally, the log file records all user actions, from querying the AOP-Wiki RDF to exporting to Cytoscape, ensuring transparency and reproducibility. It serves as a valuable record, guiding users through their interactions and any transformations applied to the AOP graph.

## 4 Discussion

The AOP-networkFinder employs a multi-faceted approach, leveraging RDF and user-friendly interfaces to streamline data extraction, AOP network construction, and information retrieval. By addressing limitations and simplifying processes, the tool enhances the accessibility and usability of AOP content, contributing to the advancement of AOP-related research and applications. By offering a user-friendly interface, utilizing the flexibility of AOP knowledge graphs, and integration with Cytoscape, the tool distinguishes itself from existing tools like the AOP-Wiki SPARQL endpoint (Martens *et al.* 2022) by eliminating the need for SPARQL queries and complements specialized tools like AOP-helpFinder (Jaylet *et al.* 2023).

The AOP-networkFinder aligns with the FAIR principles (Wilkinson *et al.* 2016) by enhancing the accessibility of AOPs and AOP networks from AOP-Wiki and contributing to the findability and reusability of AOP-related information. The seamless integration with Cytoscape also allows for further network exploration and analysis. The tool significantly improves accessibility for toxicologists to extract, visualize, and manipulate AOPs and AOP networks, reducing barriers to utilizing the AOP-Wiki’s data. This accessibility is crucial for fostering collaboration and knowledge-sharing among toxicology experts. Furthermore, by expanding KEs with genes via the AOP-Wiki RDF, the tool enhances the integration of omics data with the AOP, which is essential to facilitate the uptake of omics data for regulatory purposes. Additionally, the nearest neighbour method has the potential to establish novel connections that scientists may not have envisioned, fostering innovation. Nevertheless, the utilization of both displaying genes and the nearest neighbour approach requires careful consideration and curation, as they may inadvertently produce artefacts.

Allowing users to merge KEs based on their functional similarity addresses a critical curation need within the AOP-Wiki, enhancing the quality of AOP networks and fostering collaboration within the toxicology community. However, the lack of a method to push changes into the AOP-Wiki directly from third-party tools such as the AOP-networkFinder poses a challenge for such curation efforts. To facilitate external curation of AOP-Wiki content, it would be beneficial to implement version-controlled AOPs, accompanied by clear curation guidelines. This would allow community edits that require author approval before being incorporated into the AOP-Wiki. Another limitation involves inaccurately submitted AOPs to AOP-Wiki. Through utilization of the tool in PARC, numerous instances of the same KEs, missing KERs in a machine-readable format, and poorly developed AOPs were observed, calling for improvements in original AOPs. Although the tool includes a filter for approved AOPs, this significantly limits the utility as the tool is based on existing data provided by researchers.

The AOP-networkFinder effectively utilizes KERs as functional units, enabling the construction of AOP networks. Its ability to automatically identify and merge similar KEs enhances the connectivity, providing a holistic view of relationships between MIEs and AOs. While this aligns with the core principles of the AOP framework, reliance on user judgment for merging KEs based on name similarity is a limitation. Although this tool provides AI-suggested KE merges, the user must validate these (based on expert knowledge). Addressing this limitation may involve incorporating more advanced algorithms or providing additional information for users, with Natural Language Processing (NLP) tools potentially enhancing the assessment and categorization of comparable KEs.

The modular structure of the AOP-networkFinder allows for the seamless addition of new features. Future iterations could explore incorporating advanced search functionalities, such as semantic search or machine learning algorithms for automatic and more extensive KE similarity assessments. These developments will enhance the tool’s capacity for more complex tasks, such as more accurate KE merging based on NLP and more precise biological pathway analysis. In addition, incorporating quantitative metrics for assessing the quality and completeness of AOP networks would further improve its scalability, allowing researchers to evaluate networks with more depth and precision. As the tool evolves, these features will increase its usability across different research domains, making it a versatile resource for both novice and expert users. This tool is also a starting point for quantitative AOP development, if quantitative data for KERs are available. Collaboration within the AOP community is essential for ongoing refinement, ensuring that the tool aligns with evolving community needs and standards.

The AOP-networkFinder enhances AOP content accessibility and empowers user-driven curation of AOP networks. With possibilities for new features, user-driven curation, and consideration of limitations, the tool represents a significant step toward advancing the understanding and application of AOPs in risk and hazard assessments.

## Supplementary Material

vbaf007_Supplementary_Data

## Data Availability

The source code can be found in the Zenodo community ‘Computational Toxicology at Norwegian Institute of Public Health’ with doi 10.5281/zenodo.11068434 and in the GitHub at github.com/folkehelseinstituttet/AOPnetworkFinder_v1. The tool extracts AOP information through the AOP-Wiki SPARQL endpoint. A prebuilt Docker image is available at hub.docker.com/r/nurre123/aop_network_finder. The software is available under the GNU Affero General Public License (AGPL), version 3.0.
