# Supplementary material for: AOP-networkFinder—a versatile tool for the reconstruction and visualization of Adverse Outcome Pathway networks from AOP-Wiki
Source: Bioinform Adv. 2025 Jan 22;5(1):vbaf007. doi: 10.1093/bioadv/vbaf007 (PMC11835234; doi:10.1093/bioadv/vbaf007)
Supplement: vbaf007_Supplementary_Data [file vbaf007_supplementary_data.zip › de896_R2_Supplementary_AOP-networkFinder.docx]

**Supplementary Online Appendix**

**AOP-networkFinder - A versatile tool for the reconstruction and visualization of Adverse Outcome Pathway networks from AOP-Wiki**

Nurettin Yarar ^1,2^, Marvin Martens ^3^, Torbjørn Rognes ^1,4^, Jan Lavender ^5^, Hubert Dirven ^2^, Karine Audouze ^6^, Marcin W. Wojewodzic ^2,7 #^

# Corresponding author [Marcin.Wojewodzic@fhi.no](mailto:Marcin.Wojewodzic@fhi.no), ORCID: 0000-0003-2501-5201

Associations:

1. Department of Informatics, University of Oslo, Oslo, Norway
2. Department of Chemical Toxicology, Norwegian Institute of Public Health, Norway
3. Department of Bioinformatics (BiGCaT), NUTRIM, Faculty of Health, Medicine and Life Sciences, Maastricht University, Maastricht, The Netherlands
4. Department of Microbiology, Oslo University Hospital, Oslo, Norway
5. Department Computer Science, University of East Anglia, United Kingdom
6. University Paris Cité, Inserm T3S, Paris, France
7. Department of Research, Cancer Registry of Norway, Norwegian Institute of Public Health, Oslo, Norway

##
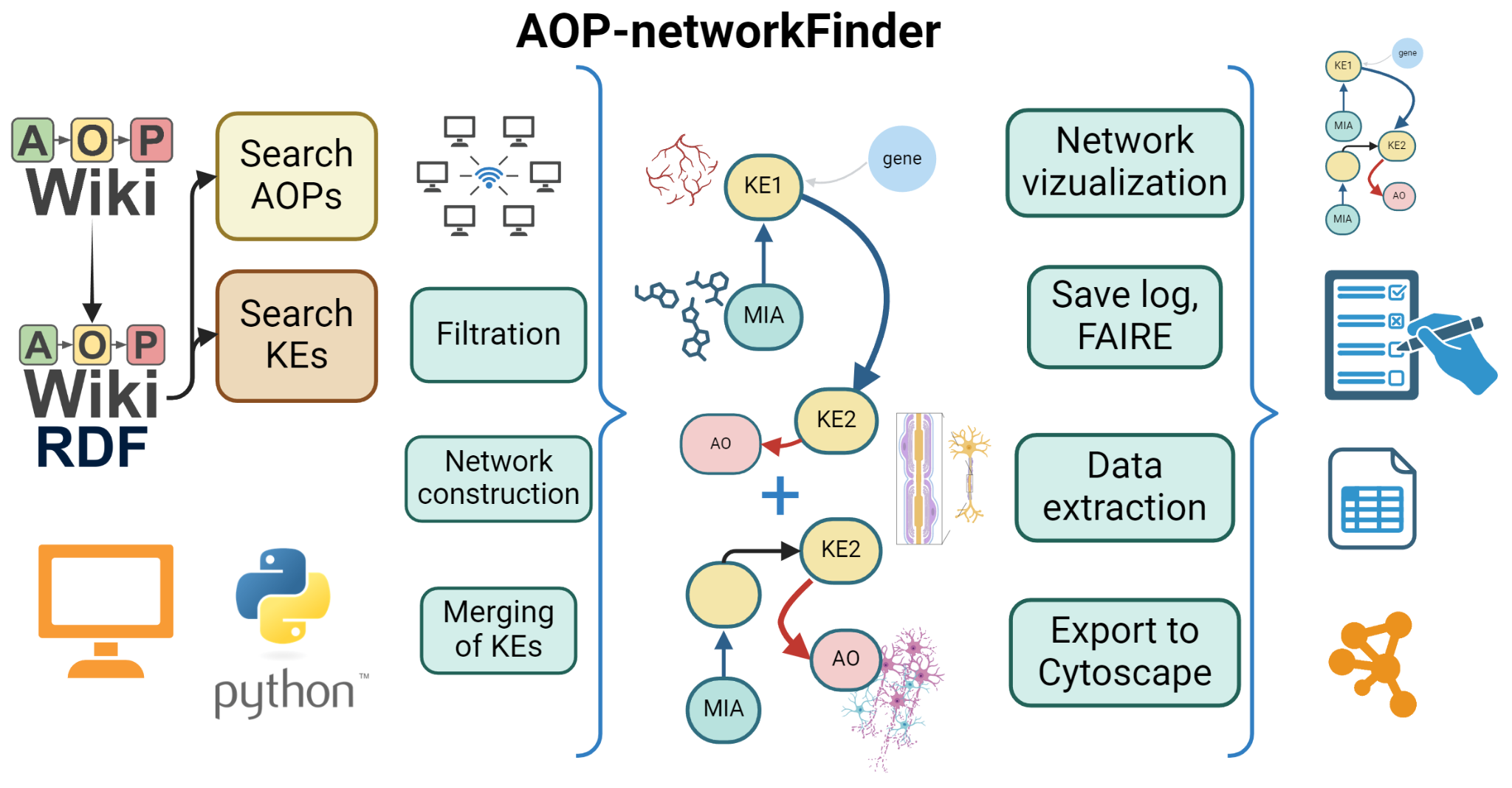


Created with BioRender.com


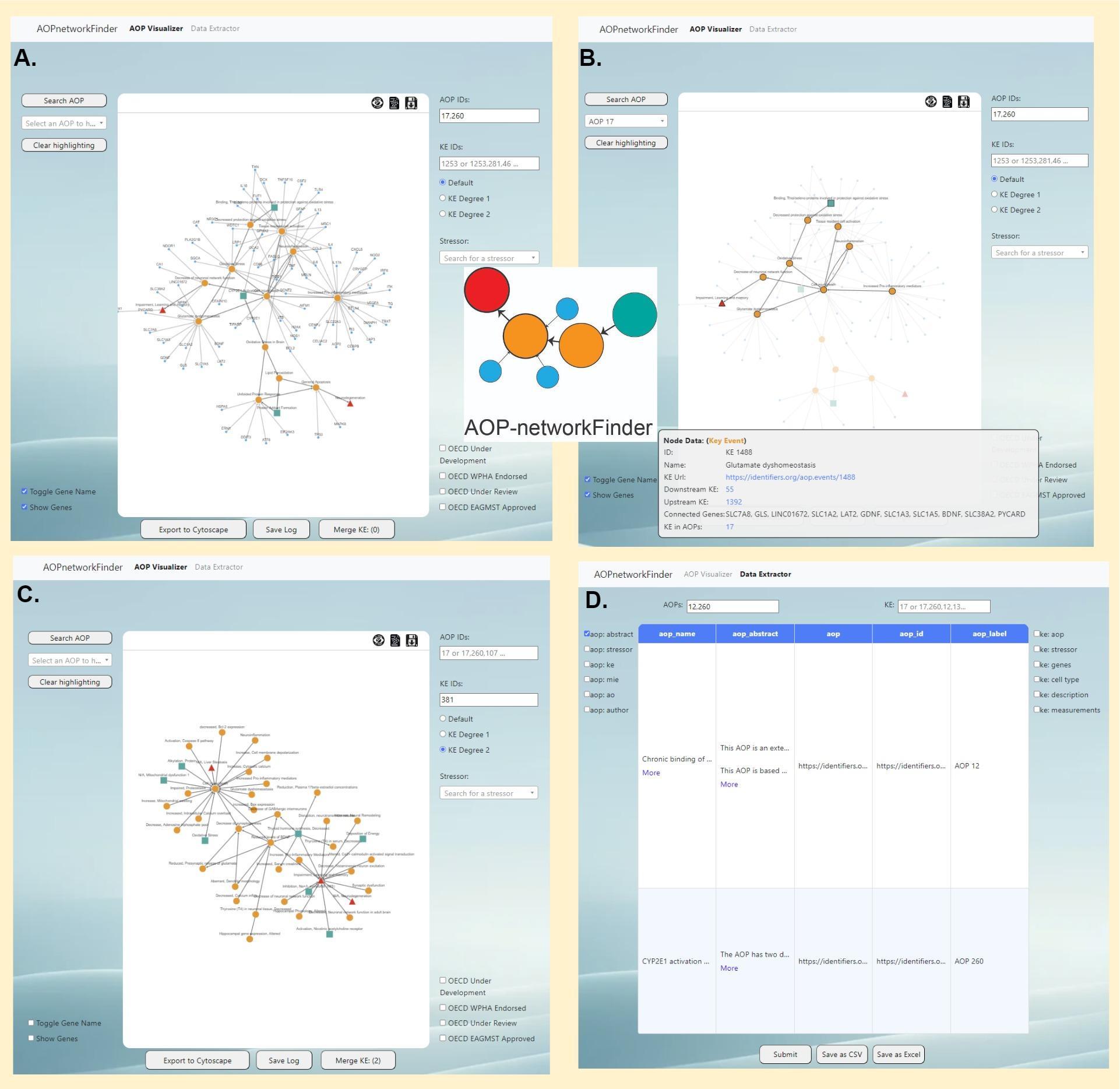


**Supplementary Figure 1.**  Main functionalities implemented in AOP-networkFinder. Green squares represent molecular initiation events (MIEs) in the AOP, orange circles represent adherent Key Events and red triangles represent Adverse Outcome (AO), at the end of the path. The arrows illustrate the direction of Key Events Relationship (KER). Blue circles represent genes connected to KEs. A. Reconstruction of AOP network based on AOP ID given by user. There is a possibility for displaying genes associated with KE as well as filtration of AOPs ID according to its degree of endorsement (OECD). The reconstructed network can further be exported to jpg/png or Cytoscape. B. Displaying information about the KE or gene in relation to the whole network is possible, as well as addition of non-existing connections. Highlighting of given AOP is unable. C. Given degree from KEs there is a possibility to reconstruct the network around this given KEs. D. Data extraction in forms of csv and excel format from AOP-Wiki for chosen AOPs and KEs.

**Supplementary Table 1:** Comparison of AOP-networkFinder tool with some other tools for processing or reconstruction of AOPs. Notice that list is non-exhaustive.

| **Tool/Platform** | **Location/Link** | **Functionalities** | **Interface type** | **Data access method** | **Need for SPARQL knowledge** | **Ease of use** | **AOP network reconstruction** | **Main advantages** | **Output/Export options** |
| --- | --- | --- | --- | --- | --- | --- | --- | --- | --- |
| AOP-NetworkFinder | https://aop-networkfinder.no/ | AOP network reconstruction and visualization | Web | SPARQL endpoint | No | Beginner/intermediate | Yes (customizable) | Automated network creation; GUI | Cytoscape, CSV, Image |
| AOP-Wiki website | https://aopwiki.org/ | AOP data browsing and contribution | Web | Direct browsing | No | Beginner | No | Comprehensive AOP repository | Web view only |
| AOP-Wiki SPARQL endpoint | https://openrisknet.org/e-infrastructure/services/133/ | Advanced data extraction and custom querying | Programming | SPARQL endpoint | Yes | Expert | Limited (requires coding) | Highly customizable | Query-based (custom formats) |
| Wiki Kaptis | https://wikikaptis.lhasacloud.org/#/aop | Producing grafs | Web | Browsing | No | Beginner | Yes, limited functionality | User friendly | None |
| sAOP | [saop.cpr.ku.dk](http://saop.cpr.ku.dk/) | Linking | Web | Browsing | No | Beginner/intermediate | Yes, limited functionality | User friendly | None |
| AOPKB – Biovista Vizit | https://aopkb.biovista.com/ | Connecting AOPs via Kes; Explore and create interactive networks | Web | Browsing | No | Beginner/intermediate | Yes, limited functionality | User friendly | HTML, PNG, SVG |
| AOP-helpFinder | https://aop-helpfinder.u-paris-sciences.fr/ | Development of new AOP using abstracts in Pubmed | Web | Registration required; data given in TXT files | No | Advance | No | Supporting Key Events relationship | Zip with CSV, HTML, Image |
| AOPWIKI-Explorer | https://github.com/Crispae/AOPWiki_Explorer | AOP construction based on NLM | GitHub, Web (not working by 2.11.2024) | Accessing XML format | No | Beginner | Yes (unsupervised) | Automated quick overview | Image |
